# Prognostic Value of Nutritional Risk Scores in Septic ICU Patients: A Survival Analysis Using mNUTRIC, PNI, and CONUT

**DOI:** 10.3390/diagnostics16081193

**Published:** 2026-04-16

**Authors:** Marius Bogdan Novac, Gabriel-Petre Gorecki, Alin Pătru, Anda Lorena Dijmărescu, Diana-Ruxandra Hădăreanu, Mohamed-Zakaria Assani, Lidia Boldeanu, Mihail Virgil Boldeanu, George Alin Stoica

**Affiliations:** 1 Department of Anesthesiology and Intensive Care, University of Medicine and Pharmacy of Craiova, 200349 Craiova, Romania; marius.novac@umfcv.ro; 2 Department of Anesthesia and Intensive Care, Faculty of Medicine, “Titu Maiorescu” University, 031593 Bucharest, Romania; gabriel.gorecki@prof.utm.ro; 3 Department of Anesthesiology and Intensive Care, Clinical Emergency County Hospital of Craiova, 200642 Craiova, Romania; alin_tz@yahoo.com; 4 Department of Obstetrics and Gynecology, Faculty of Medicine, University of Medicine and Pharmacy of Craiova, 200349 Craiova, Romania; lorenadijmarescu@yahoo.com; 5 Department of Cardiology, University of Medicine and Pharmacy of Craiova, 200349 Craiova, Romania; diana.hadareanu@umfcv.ro; 6 Doctoral School, University of Medicine and Pharmacy of Craiova, 200349 Craiova, Romania; mohamed.assani@umfcv.ro; 7 Department of Microbiology, Faculty of Medicine, University of Medicine and Pharmacy of Craiova, 200349 Craiova, Romania; 8 Department of Immunology, Faculty of Medicine, University of Medicine and Pharmacy of Craiova, 200349 Craiova, Romania; 9 Department of Pediatric Surgery and Orthopedics, Faculty of Medicine, University of Medicine and Pharmacy of Craiova, 200349 Craiova, Romania; alin.stoica76@gmail.com

**Keywords:** malnutrition, sepsis, adverse outcome, critically ill, mNUTRIC score, PNI score, CONUT score, ICU

## Abstract

**Background**: Malnutrition is highly prevalent among critically ill patients and has been associated with worse clinical outcomes, particularly in sepsis. Several nutritional risk scores have been proposed to identify patients at increased risk of mortality in the intensive care unit (ICU). This study aimed to evaluate the prognostic value of three commonly used nutritional indices—modified Nutrition Risk in the Critically Ill (mNUTRIC), Prognostic Nutritional Index (PNI), and Controlling Nutritional Status (CONUT)—for predicting mortality in septic ICU patients. **Methods**: In this prospective observational cohort study conducted at two ICUs, 155 critically ill patients at nutritional risk were evaluated, including 105 patients with sepsis and 50 without sepsis. The primary endpoint was ICU mortality. Nutritional risk scores (mNUTRIC, PNI, and CONUT) were calculated at ICU admission. Survival analysis was performed using Kaplan–Meier (KM) curves and log-rank tests to compare survival probabilities across nutritional risk categories. Cox proportional hazards regression analysis was used to assess the association between nutritional scores and ICU mortality. Of note, only 24 mortality events were recorded in the septic cohort, which limits the statistical power of the findings. **Results**: KM analysis revealed significantly reduced survival among patients with severe malnutrition, as measured by the PNI score (log-rank *p* = 0.044). Patients with high mNUTRIC scores showed a tendency toward lower survival probability compared with those with low nutritional risk, approaching statistical significance (log-rank *p* = 0.059). No significant survival differences were observed between CONUT categories (log-rank *p* = 0.380). In univariate Cox regression analysis, the mNUTRIC score was significantly associated with ICU mortality (HR 1.67, 95% CI 1.17–2.38, *p* = 0.005). **Conclusions**: In this selected cohort, mNUTRIC demonstrated the strongest univariate prognostic signal for ICU mortality; however, this association was attenuated and did not reach statistical significance after limited multivariable adjustment. These findings are exploratory and apply specifically to a cohort of septic ICU patients with confirmed nutritional risk and therefore should not be generalized to the broader population of critically ill septic patients.

## 1. Introduction

Sepsis remains one of the leading causes of Intensive Care Unit (ICU) admission worldwide, with mortality rates exceeding 25% even in high-income settings [1]. It is characterized by a dysregulated host response to infection, involving both an early systemic inflammatory response and a concomitant immunosuppressive state that may persist throughout the disease course. This complex pathophysiology contributes to the development of organ dysfunction, particularly in vulnerable populations such as elderly patients [2]. Malnutrition is highly prevalent among ICU patients and is associated with adverse clinical outcomes, including increased mortality, higher complication rates, and prolonged hospitalization. Previous studies have reported that the prevalence of malnutrition in critically ill patients ranges from 38% to 78%, underscoring its clinical significance [3]. In the context of sepsis, nutritional assessment is particularly challenging due to the profound inflammatory response and associated metabolic alterations. Serum biomarkers commonly used in nutritional indices, such as albumin, are significantly influenced by the acute-phase response, capillary leakage, and fluid shifts, which may limit their reliability as indicators of nutritional status in this population. Under conditions of severe metabolic stress, pro-inflammatory mediators are markedly upregulated, leading to an intense inflammatory response and accelerated tissue catabolism. This process contributes to the rapid development or worsening of malnutrition, even in patients without prior evidence of nutritional deficits. Furthermore, despite efforts to provide appropriate nutritional support, critically ill patients may still receive insufficient intake due to difficulties in accurately estimating nutritional requirements and the practical challenges of delivering adequate nutrition in the ICU setting. This consideration underscores the importance of early, optimized nutritional support in critically ill patients to mitigate the adverse effects of malnutrition on clinical outcomes and healthcare resource utilization. Further research is needed to develop and implement effective strategies for the prevention and management of malnutrition in this vulnerable population [4,5].

Guidelines from the European Society of Clinical Nutrition and Metabolism (ESPEN) recommend systematic nutritional evaluation of patients to identify those who are malnourished or at risk of malnutrition [6]. Routine screening for malnutrition risk in patients admitted to the ICU is essential, as it enables early identification of individuals who may benefit from targeted nutritional interventions [7]. Such screening should be performed within the first 24–48 h of ICU admission. According to ESPEN guidelines, critically ill patients who require ICU care for more than 48 h are at particularly increased risk of developing malnutrition [8].

The assessment of nutritional status can currently be performed using a variety of scoring systems and tools designed to evaluate both nutritional and immunological status. However, most of these instruments were originally developed for hospitalized patients outside the ICU setting. In 2011, Heyland et al. highlighted that not all critically ill patients with nutritional impairment are at equal risk of adverse clinical outcomes. Based on this concept, the Nutrition Risk in the Critically Ill (NUTRIC) score was developed and validated specifically for ICU patients, allowing the identification of individuals at higher nutritional risk who may benefit from aggressive nutritional therapy [9]. The Prognostic Nutritional Index (PNI), originally introduced by Onodera et al. [10] to assess the risk of postoperative complications in patients with gastrointestinal malignancies, was subsequently extended to other clinical conditions [11]. This index reflects both nutritional and inflammatory status. The Controlling Nutritional Status (CONUT) score was later proposed as a simple and cost-effective tool for assessing malnutrition in hospitalized patients [12].

CONUT and PNI are practical nutritional indicators widely used in clinical settings, as they are based on parameters routinely included in standard patient investigations. However, albumin-based tools rely on serum albumin, either alone or as part of composite indices, as a surrogate marker of nutritional reserve. This reliance is particularly problematic in sepsis, where the acute-phase response induces a rapid, inflammation-driven decrease in serum albumin levels through increased vascular permeability, redistribution to the extravascular space, and hepatic reprioritization toward the synthesis of positive acute-phase proteins. In this context, hypoalbuminemia reflects systemic inflammation rather than true nutritional depletion, potentially confounding albumin-based risk stratification and leading to an overestimation of malnutrition prevalence in a population where it is already high. These indices are based on routinely measured parameters, including serum albumin, total cholesterol, and peripheral lymphocyte counts. Nevertheless, current evidence regarding the prognostic performance of PNI and CONUT in ICU patients remains limited and, in some cases, conflicting [13].

The NUTRIC score was developed to assess nutritional risk in critically ill patients, particularly those with poor nutritional status who are at increased risk of adverse clinical outcomes in the ICU. It is based on a multidimensional model that integrates variables reflecting both disease severity and nutritional status, including markers of inflammation, organ dysfunction, and comorbidity burden. The components of the NUTRIC score include age, the Acute Physiology and Chronic Health Evaluation (APACHE II), the Sepsis-related Organ Failure Assessment (SOFA) scores, the number of comorbidities, the interval between hospital admission and ICU admission, and interleukin 6 (IL-6) levels. A modified version of the score (mNUTRIC), which excludes IL-6, was subsequently validated in 2015 and is more commonly used in clinical practice [14].

Approximately a decade ago, the ESPEN Guidelines on Parenteral Nutrition in Critical Care recommended the use of artificial nutrition, particularly in patients at high nutritional risk, thereby emphasizing the importance of systematic nutritional risk assessment [15]. However, subsequent studies in critically ill populations have suggested that early aggressive artificial feeding may not provide any benefit and could even be associated with harm [16,17,18,19]. These findings have contributed to a shift toward a more conservative approach to artificial nutrition during the early phase of critical illness, particularly within the first week of ICU admission, as reflected in more recent European recommendations [20].

Sepsis remains a major global health concern in critically ill patients, with high mortality rates further exacerbated by malnutrition. As a complex host response to infection, it has an impact reflected in its global burden: approximately 1.7 million cases annually in the United States [21], one-third of ICU admissions in the United Kingdom [22], and nearly one-fifth of critically ill patients in China [23]. In 2017, an estimated 48.9 million sepsis cases and 11 million deaths were reported worldwide, although recent trends suggest a gradual decline, partly attributed to initiatives such as the Surviving Sepsis Campaign and advances in sepsis definitions, including Sepsis-3 [1,24,25,26].

The severe inflammatory response and associated metabolic stress in septic patients contribute to accelerated tissue catabolism and impaired immune function, helping to explain both the high prevalence of malnutrition in the ICU and its impact on increased susceptibility to infection; accordingly, malnutrition has been described as the “skeleton in the hospital closet,” reflecting its frequent under-recognition, underestimation, and undertreatment, which may lead to adverse and potentially irreversible outcomes [27].

Therefore, we conducted a prospective observational study in adult patients admitted to the ICU with a primary diagnosis of sepsis and at risk of malnutrition. We hypothesized that the mNUTRIC score would provide superior prognostic discrimination for 28-day ICU mortality compared with albumin-based indices (PNI and CONUT). Secondary endpoints included length of stay in the ICU (LOS-ICU) and hospital (LOS-H), as well as the need for mechanical ventilation.

## 2. Materials and Methods

### 2.1. Data Sources

We conducted an observational, non-invasive, prospective cohort study between January 2024 and December 2024 at two ICUs: the Clinical Municipal Hospital “Filantropia” Craiova (Ethics Committee approval no. 886, 15 January 2024) and the Emergency County Clinical Hospital of Craiova (Ethics Committee approval no. 2371, 14 January 2022). This report follows the STROBE (Strengthening the Reporting of Observational Studies in Epidemiology) guidelines. Data collection was prospective; the study is therefore correctly classified as a prospective observational bicentric cohort study throughout this manuscript. This report follows the STROBE (Strengthening the Reporting of Observational Studies in Epidemiology) guidelines.

During this study period, 332 septic patients admitted to the ICUs of the Clinical Municipal Hospital Filantropia and the Emergency County Clinical Hospital of Craiova, Dolj, were reviewed and classified according to the inclusion/exclusion criteria. We only included patients at risk of malnutrition based on their mNUTRIC, PNI, and CONUT scores. We selected 155 patients with malnutrition, 105 with sepsis, and 50 without sepsis who met the inclusion criteria and signed the Free and Informed Consent Form before evaluation.

### 2.2. Eligibility Criteria

Inclusion criteria were: patients aged 18 years or older, admitted to the ICU within 72 h, and at risk of malnutrition, as confirmed by all three nutritional scoring tools (PNI, CONUT, and mNUTRIC) simultaneously. This convergent multi-score approach was chosen to ensure that all enrolled patients had clear nutritional risk across independent assessment frameworks. The authors acknowledge that this design introduces a selection effect: by pre-selecting patients using the very instruments under investigation, score distributions are necessarily restricted, and apparent prognostic performance may differ from that observed in unselected ICU populations. Results should therefore be interpreted as applicable to patients with confirmed nutritional risk rather than generalized to all septic ICU admissions.

Patients who refused to participate were excluded. Patients who were unable to provide direct informed consent due to sedation, intubation, or altered mental status were enrolled only after obtaining written informed consent from a legally authorized representative (next of kin or designated proxy), in accordance with the Declaration of Helsinki and Romanian regulations on emergency research involving incapacitated adults. A total of 12 eligible patients were excluded because neither the patient nor a proxy representative was available to provide consent within the required timeframe. This may have introduced a degree of selection toward less severely ill patients, which is acknowledged as a limitation.

### 2.3. Variables and Measurement

Patients admitted to the ICU were assessed for nutritional status using three nutritional screening tools and explanatory variables both at admission and on the third day.

On the third day, we utilized the secondary assessment because we considered that patients still had a significant risk for complications after three days in the intensive care unit and that determining nutritional status is essential to ensuring adequate nutrition.

All patients in the study received standard treatment for sepsis or septic shock in accordance with the SSC International Guidelines for the Management of Sepsis and Septic Shock [25,28].

#### 2.3.1. Explanatory Parameter

Several variables were evaluated, including sex, age, body mass index, residence, sepsis etiology, length of stay in the ICU, mechanical ventilation, mortality, and comorbidities. To follow the evolution of the cases, several laboratory data were considered: complete blood count (FBC), blood urea nitrogen (BUN), creatinine, total cholesterol, lactate dehydrogenase (LDH), and other biochemical and coagulation parameters. All patients were checked upon admission to the ICU, during the acute inflammatory phase, and again three days later, after the acute phase, when a more stable catabolism is estimated.

#### 2.3.2. Nutritional Status Assessments

We assessed the risk of malnutrition at ICU admission and on the third day using the PNI, CONUT, and mNUTRIC scores.

The formula for calculating the PNI score was 10 × serum albumin (g/dL) + [0.005 × absolute lymphocyte count (per mm^3^)]. Depending on the PNI score, patients’ nutritional status was classified as normal (PNI > 50), mild or moderate malnutrition (PNI 45–50 and 40–45), or severe malnutrition (PNI < 40) [29,30]. For this analysis, given the limited number of patients with mild malnutrition (PNI 45–50), patients were grouped into two clinically relevant categories: moderate (PNI 40–45) and severe (PNI < 40). Patients with normal status (PNI > 50) were few and were included in the moderate category for comparative analyses.

The CONUT score is calculated as the serum albumin score + the total cholesterol score + the total lymphocyte score. Each parameter receives a score, and these scores are summed to produce an overall CONUT score. Of the total 12 points in the CONUT score, a score above 5 indicates malnutrition [31].

Since IL-6 measurement is not routinely available in our institution, the mNUTRIC score was applied, excluding IL-6 from the original NUTRIC formula. The mNUTRIC has been validated as a reliable surrogate with comparable predictive accuracy [13,14]. The mNUTRIC is a composite score that incorporates age, APACHE II score, SOFA score, number of comorbidities, and the number of days from hospital admission to ICU admission. Because APACHE II and SOFA are structural components of the mNUTRIC formula, including both simultaneously in a regression model introduces structural collinearity; Cox and logistic regression models were therefore built with mNUTRIC adjusted only for age. Patients with a score ≥ 6 are considered to have a high nutritional risk, associated with a prolonged ICU stay and increased mortality [32]. We calculated the mNUTRIC score using a validated online calculator available at MDCalc [33].

The SOFA and APACHE II scores were used to assess disease severity at admission and again on the third day. The SOFA score was utilized to evaluate the severity of the patient’s condition within the first 24 h of admission to the intensive care unit. A SOFA score > 11 indicates 100% mortality, while lower scores have varying degrees of survival. SOFA was initially developed to assess the severity of organ dysfunction in sepsis, but it was also used to determine the severity of organ dysfunction in critically ill patients in the ICU and has recently been used as an indicator of mortality in the ICU [34].

When determining the severity of a patient’s disease, the APACHE II score is a useful predictor. In all patients admitted to the ICU, the APACHE II and SOFA scores were calculated within the first 24 h. On the third day, we re-evaluated these scores.

#### 2.3.3. Ethical Considerations

The study was conducted in accordance with the Declaration of Helsinki and was approved by the Ethics Committee of the Clinical Municipal Hospital Filantropia (no. 886/15 January 2024) and the Emergency County Clinical Hospital of Craiova (no. 2371/14 January 2022), Dolj, Romania.

#### 2.3.4. Statistical Analysis

We processed and handled patient data from medical records using Microsoft Excel. To analyze the data, we utilized GraphPad Prism 11.0.0 Version (GraphPad Software, San Diego, CA, USA). The data were examined for normality using the Shapiro–Wilk and Kolmogorov–Smirnov tests.

The clinical and demographic characteristics of the study participants were compiled using descriptive statistics. The variables are presented as the mean and standard deviation (SD) for variables that had normal distributions and as the median with an interquartile range for variables that had non-normal distributions. Frequencies and percentages were used to represent categorical variables.

Continuous variables were evaluated using Student’s *t*-test/One-Way ANOVA or the Mann–Whitney test/Kruskal–Wallis’s test (used for non-Gaussian distributions) to find the difference between groups, and the χ2 test was used for categorical variables.

To check for any significant correlations between the levels or values of the parameters, Spearman’s coefficients (−1 < rho < 1) were used.

ROC curve analysis was used to evaluate the discriminative capacity of the nutritional scores for ICU hospitalization outcomes and length of stay at two fixed time points (Day 1 and Day 3). For the primary time-to-event mortality endpoint, time-dependent ROC or concordance statistics (Harrell’s C-statistic) would be methodologically preferable; however, given the limited number of mortality events (*n* = 24), conventional ROC analysis was applied as an exploratory approach for the mortality endpoint as well, and results should be interpreted with appropriate caution. We quantified performance using the AUC and p-statistics, comparing the calculated AUC against a threshold of AUC = 0.5, which indicates a weak discriminative marker. To identify the cut-off values that yielded maximum accuracy, we calculated the sensitivity, specificity, and Youden index (sensitivity + specificity − 1) for each marker at the different threshold values investigated.

Survival analysis was performed to evaluate the association between nutritional risk scores and ICU mortality in patients with sepsis. Time-to-event was defined as the interval between ICU admission and death or censoring during the observation period. Kaplan–Meier survival curves were constructed to estimate survival probabilities according to nutritional risk categories defined by the mNUTRIC, PNI, and CONUT scores, and differences between groups were assessed using the log-rank test. In addition, Cox proportional hazards regression analysis was performed to evaluate the association between clinical and nutritional variables and the risk of ICU mortality. Hazard ratios (HRs) and 95% confidence intervals (CIs) were calculated. Because age, APACHE II, and SOFA scores are structural components of the mNUTRIC index, these variables were excluded from multivariable models to avoid structural collinearity. Therefore, in addition to univariate Cox analyses, an adjusted Cox proportional hazards model including mNUTRIC and variables not incorporated into the score (sex and mechanical ventilation) was constructed. The proportional hazards assumption was assessed graphically using log–log survival plots; no material violations were identified. Day-3 analyses should be interpreted with caution due to potential survivor bias, as only patients surviving to Day 3 were reassessed. A *p*-value < 0.05 was considered statistically significant.

## 3. Results

### 3.1. The Clinical and Demographic Characteristics of People with Sepsis and Nonsepsis

As presented in Table 1, this study included 105 patients diagnosed with sepsis (S), aged 30–83, with a mean ± SD age of 62.56 ± 11.78. Most patients were males (61.90%, 65 males and 40 females). In the control, non-septic (NS) group, the mean ± SD age was 59.74 ± 14.30, with females comprising 54.00%. No significant difference was found between the two study groups.

Thus, no statistically significant differences were observed regarding age (*p* = 0.318) and gender (χ^2^(1) = 0.150, *p* = 0.062). Regarding where the patients lived, most patients in the S and NS groups (54 and 36, respectively) were from urban areas; there was a statistically significant difference between the two groups (χ^2^(1) = 0.190, *p* = 0.015).

In our study, 98 patients with S had 2 or multiple comorbidities: diabetes mellitus (DM), vascular (hypertension, HTN), myocardial (angina, arrhythmia, heart disease, myocardial infarction (MI)), musculoskeletal (arthritis, osteoporosis, connective tissue disease), hemodynamic instability, and pulmonary comorbidities (asthma, chronic obstructive pulmonary disease (COPD)). In the NS group, 36 patients had 2 or multiple comorbidities.

Analyzing the severity of the patient’s disease, our study showed that patients with S have statistically significant differences among APACHE II [28 (15–44) vs. 18 (8–35), *p* < 0.0001] and SOFA [9 (4–13) vs. 7 (2–11), *p* < 0.0001] scores.

The groups were not comparable in terms of the reasons for ICU admission.

A statistically significant difference was noted between the two groups (S vs. NS) regarding laboratory parameters: LYM (*p* = 0.0006), PLT (*p* < 0.0001), procalcitonin (*p* < 0.0001), ALB (*p* = 0.038), CRP (*p* = 0.027), TC (*p* = 0.038), and WBC (*p* = 0.053), with the latter reaching the significance limit. According to our results, S patients exhibit a poorer inflammatory and nutritional status. This observation is supported by statistically significant differences in nutritional-inflammatory status between S and NS patients, as measured by mNUTRIC [7 (3–9) vs. 5 (1–8), *p* < 0.0001]. For the PNI [38.95 (14.00–55.85) vs. 36.50 (21.50–52.50), *p* = 0.345] and CONUT [5 (2–12) vs. 6 (2–12), *p* = 0.136] indices, our study found no statistically significant differences between the groups.

According to PNI, 61.90% (*n* = 65) of the S population was at risk of severe malnutrition, with no significant difference between the S and NS groups (*p* = 0.862). Similarly, mNUTRIC indicated that 74.28% (*n* = 78) of the S group had high malnutrition risk, which was significantly higher than in the NS group (*p* = 0.011). According to the CONUT, 54.28% (*n* = 57) of the S population had a severe risk of malnutrition, which was statistically significant compared with the NS group (*p* = 0.0002).

The groups differ in terms of median LOS-ICU (*p* < 0.0001) and LOS-H (*p* < 0.0001). LOS-ICU and LOS-H were significantly higher in the S group [8 (4–15) days vs. 5 (3–10) days; 16 (9–24) days vs. 8 (3–20) days].

Our study revealed that 43 patients with sepsis required mechanical ventilation (MV), which was significantly more frequent in the septic group than in non-septic patients (*p* < 0.0001), consistent with the higher severity of illness in the sepsis cohort.

The analysis conducted on the third day of ICU admission showed that patients with S had statistically significant differences in disease severity, as assessed by APACHE II [25 (9–43) vs. 16 (8–35), *p* < 0.0001] and SOFA [8 (3–15) vs. 6 (3–17), *p* < 0.0001] scores (Table 2).

Significant differences between median values in subjects with S and NS were identified for WBC [11.80 (2.70–72.50) vs. 10.25 (3.20–19.90), *p* = 0.006], LYM [1.26 (0.10–7.20) vs. 0.94 (0.20–2.00), *p* = 0.046], and PLT [157.50 (38.00–309.00) vs. 236.00 (34.00–439.00), *p* < 0.0001].

No statistically significant difference in serum albumin was found between the S and NS groups on Day 3 (2.84 vs. 2.90 g/dL, *p* = 0.427). This absence of significance is consistent with the interpretation that albumin reduction in this cohort reflects acute-phase inflammatory response rather than chronic nutritional depletion. PNI showed no statistically significant differences between the S and NS groups, and patients with S had severe malnutrition on day 3 of ICU admission (78 patients, 74.28%, *p* = 0.413). Based on the mNUTRIC value, an appropriate nutritional assessment tool for critically ill patients, we found that 55.24% of S patients were at high risk of malnutrition (*p* = 0.025).

On the third day of ICU admission, 58 (55.24%) patients with sepsis required MV, which was significantly more frequent in the septic group than in non-septic patients (8/50, 16.0%; *p* < 0.0001), reflecting greater severity and organ dysfunction burden in the sepsis cohort. There are significant associations between the need for mechanical ventilation and nutritional-inflammatory status in patients with sepsis. This relationship is multifaceted and clinically important. Patients with sepsis who have poor nutritional status (malnutrition, low protein reserves, micronutrient deficiencies) often have compromised respiratory muscle function, including the diaphragm. This nutritional compromise can reduce respiratory muscle strength and endurance, increasing the likelihood of respiratory failure requiring mechanical ventilation.

### 3.2. Comparing Clinical Parameters Between Survivor and Non-Survivor Patients

Twenty-four patients died during hospitalization. As shown in Table 3, non-survivors were older than survivors (*p* = 0.022), with no significant differences between males and females (*p* = 0.305) or between residence (urban vs. rural, *p* = 0.120). Compared with survivors, non-survivor patients had lower ALB (*p* = 0.025), higher CRP (*p* = 0.038), and slightly higher LYM (*p* = 0.019) levels.

The APACHE II and SOFA values of the non-survivor group were significantly higher [29.50 (18–37) vs. 27.00 (15–44), *p* < 0.003, and 10 (4–14) vs. 9 (4–13), *p* = 0.046, respectively].

Although the groups did not differ in the frequency of comorbid conditions other than endocrine (survivor vs. non-survivor, *p* < 0.0001), hypertension (survivor vs. non-survivor, *p* = 0.001), myocardial (survivor vs. non-survivor, *p* = 0.002), and hemodynamic instability (survivor vs. non-survivor, *p* < 0.0001).

The groups were also comparable in terms of reasons for ICU admission (respiratory system disorders, *p* = 0.003; and trauma, *p* < 0.0001).

The groups did not differ in terms of median LOS-H (*p* = 0.361) or LOS-ICU (*p* = 0.196). The need for MV was significantly more frequent among non-survivors (*p* =< 0.0001).

Nutritional status, as assessed by PNI, revealed a severe risk of malnutrition in the study population, with a significant difference between the survivor and non-survivor groups (42 and 18 patients, respectively; *p* = 0.044). Similarly, mNUTRIC also indicated a high risk of malnutrition, with a significant difference between the survivor and non-survivor groups (56 and 22 patients, respectively, *p* = 0.027). According to the CONUT score, there was no statistically significant difference between the survivor and non-survivor groups (*p* = 0.647).

### 3.3. Comparison of the Clinical Parameters Between the PNI, mNUTRIC, and CONUT Categories on Day 1 of ICU Admission from the Sepsis Group

Comparison of the clinical parameters of the PNI, mNUTRIC, and CONUT categories on day 1 from the S group in our study revealed some particularities (Table 4).

The majority of S patients (61.90%) with severe malnutrition (PNI-severe) differed in age and laboratory parameters (*p* < 0.05). Also, these patients were associated with severe disease forms, with significantly higher APACHE II and SOFA values (*p* = 0.019 and *p* = 0.044, respectively). Additionally, S patients with PNI-severe had substantially higher median LOS-ICU (*p* = 0.046) and LOS-H (*p* = 0.039), and the need for MV was significantly more frequent (*p* = 0.0001).

Based on the mNUTRIC, patients at high risk of malnutrition differed in age (*p* < 0.0001). Regarding disease severity, S patients had significantly higher APACHE II and SOFA values (*p* < 0.0001 and *p* < 0.0001, respectively). Also, S patients with mNUTRIC-high had substantially higher median LOS-ICU (*p* = 0.034) and LOS-H (*p* = 0.026) and a significantly higher need for MV (*p* = 0.022).

### 3.4. Comparison of the Clinical Parameters Between the PNI, mNUTRIC, and CONUT Categories on Day 3 of ICU Admission from the Sepsis Group

On the third day of ICU admission, our study showed that patients at high risk of malnutrition differed in age (*p* < 0.0001) and laboratory parameters (WBC, *p* = 0.008; ALB, *p* = 0.023) only in the mNUTRIC group. Additionally, these patients had significantly higher APACHE II and SOFA scores, indicating severe disease (*p* < 0.0001 and *p* < 0.0001, respectively). Also, S patients with high mNUTRIC had substantially higher median LOS-ICU (*p* = 0.036) and LOS-H (*p* = 0.016), and the need for MV was significantly more frequent (*p* < 0.0001) (Table 5).

### 3.5. Correlations Between the PNI, mNUTRIC, CONUT, and Clinical Parameters on Day 1 of ICU Admission from the S Group

**Figure 1:**
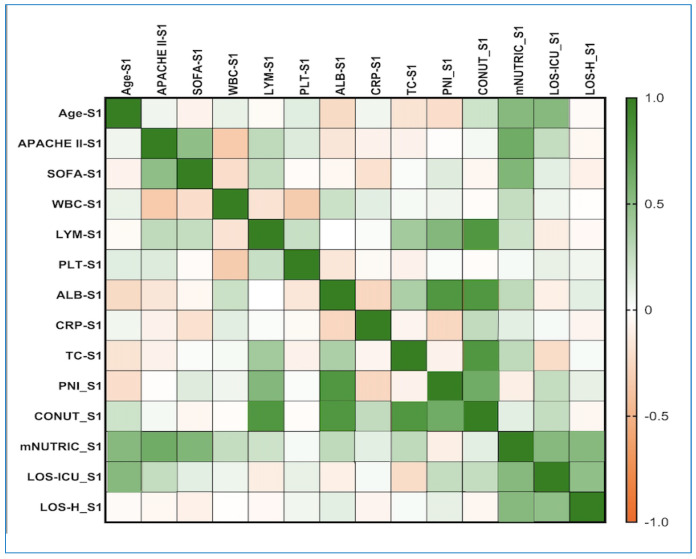
Correlations between the PNI, mNUTRIC, CONUT, and clinical parameters on day 1 of ICU admission from the S group. The correlation heatmap illustrates the relationships among the measured indicators. Strong positive correlations are shown in bright green, while strong negative correlations are shown in bright orange. APACHE II: Acute Physiology and Chronic Health Evaluation II; SOFA: Sequential Organ Failure Assessment; WBC: white blood cells/leukocytes; CRP: C-reactive protein; TC: total cholesterol; ALB: albumin; LYM: lymphocytes; PLT: platelets; PNI: Prognostic Nutritional Index; CONUT: Controlling Nutritional Status; mNUTRIC: Nutrition Risk in the Critically Ill; LOS-ICU: length of stay in Intensive Care Unit; LOS-H: length of stay in hospital.

Our study revealed that on day 1 in the ICU, among the three indices investigated, mNUTRIC (Figure 1) showed a better correlation with WBC (weak positive correlation, rho = 0.253, *p*-value = 0.009), LYM (rho = 0.213, *p*-value = 0.029), TC (rho = 0.274, *p*-value = 0.043), ALB (rho = 0.300, *p*-value = 0.001), and CRP (rho = 0.203, *p*-value = 0.015), along with the component variables of this index (age, APACHE II, SOFA, LOS-ICU). Additionally, a weak negative correlation was observed between the mNUTRIC and PNI values (rho = −0.225, *p*-value = 0.036).

### 3.6. Correlations Between the PNI, mNUTRIC, CONUT, and Clinical Parameters on Day 3 of ICU Admission from the S Group

**Figure 2:**
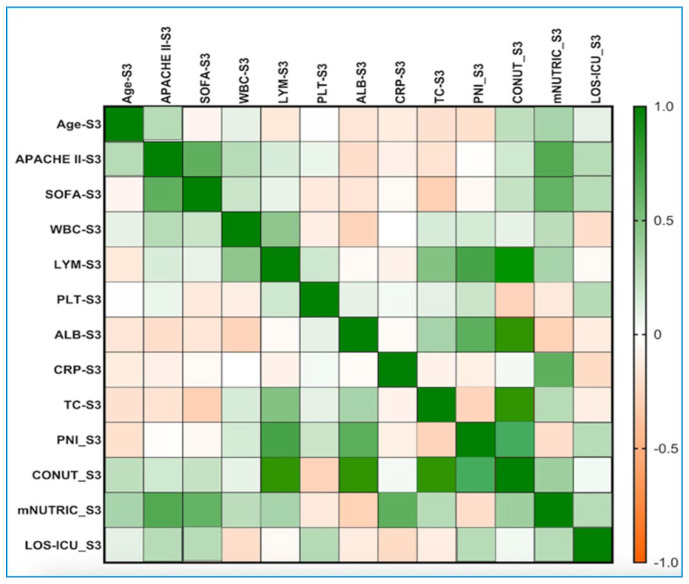
Correlations between the PNI, mNUTRIC, CONUT, and clinical parameters on day 3 of ICU admission from the S group. The correlation heatmap illustrates the relationships among the measured indicators. Strong positive correlations are shown in bright green, while strong negative correlations are shown in bright orange. APACHE II: Acute Physiology and Chronic Health Evaluation II; SOFA: Sequential Organ Failure Assessment; WBC: white blood cells/leukocytes; CRP: C-reactive protein; TC: total cholesterol; ALB: albumin; LYM: lymphocytes; PLT: platelets; PNI: Prognostic Nutritional Index; CONUT: Controlling Nutritional Status; mNUTRIC: Nutrition Risk in the Critically Ill; LOS-ICU: length of stay in Intensive Care Unit; LOS-H: length of stay in hospital.

On the third day of ICU admission (Figure 2), Spearman’s correlation analysis yielded the same results; the mNUTRIC index correlated much more strongly with clinical parameters. The mNUTRIC values exhibited weak-to-moderate positive correlations with WBC (rho = 0.267, *p*-value = 0.006), LYM (rho = 0.243, *p*-value = 0.035), CRP (rho = 0.581, *p*-value = 0.023), and TC (rho = 0.257, *p*-value = 0.014). Additionally, weak-to-moderate negative correlations were observed with ALB (rho = −0.282, *p*-value = 0.003) and PNI (rho = −0.315, *p*-value = 0.038).

### 3.7. The Accuracy of the PNI, mNUTRIC, and CONUT to Predict the Outcomes of ICU Patients

**Figure 3:**
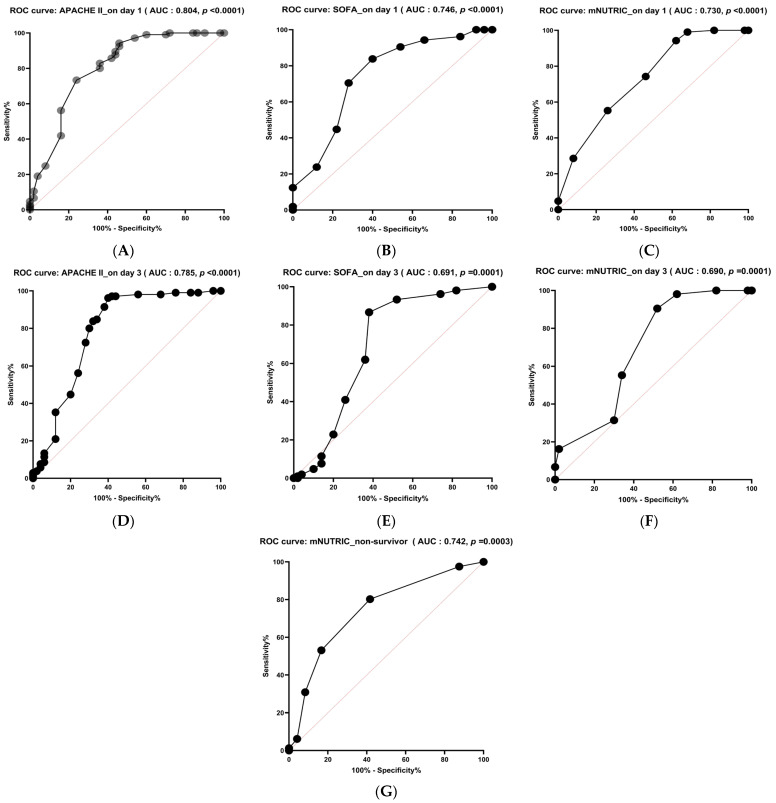
Receiver operating characteristic (ROC) curve for APACHE II on day 1 (**A**), SOFA on day 1 (**B**), mNUTRIC on day 1 (**C**), APACHE II on day 3 (**D**), SOFA on day 3 (**E**), mNUTRIC on day 1 (**F**), and mNUTRIC non-survivor (**G**).

Our study aimed to evaluate, using the ROC curve, the accuracy of the PNI, mNUTRIC, and CONUT scores in predicting ICU hospitalization outcomes, mortality, and length of stay (LOS). For each parameter, a cut-off value was determined by maximizing the sum of sensitivity and specificity. Table 6 and Figure 3 present the ROC curves for the analyzed parameters.

As we can see in Table 6, the most accurate prediction of ICU hospitalization outcomes and length of stay (LOS) was associated with the APACHE II score, both on day 1 and day 3 of ICU admission (AUC was 0.804 and 0.785, respectively), followed by the SOFA score (AUC was 0.746 and 0.691, respectively).

Of the three scores we analyzed, mNUTRIC showed the best predictive performance, both on day 1 and day 3 of ICU admission (73.00% and 69.00%, respectively).

In our study, on day 1 of ICU admission, we found the following results for indices: mNUTRIC demonstrated a sensitivity of 74.30% and a specificity of 54.00%, with an AUC of 0.730, *p* < 0.0001, and a cut-off value of 5.50; PNI exhibited a sensitivity of 60.00% and a specificity of 56.00%, with an AUC of 0.575, *p* = 0.035, and a cut-off value of 37.30.

On day 3 of ICU admission, we found the following results for indices: mNUTRIC demonstrated a sensitivity of 70.00% and a specificity of 66.00%, with an AUC of 0.690, *p* = 0.0001, and a cut-off value of 4.50; PNI exhibited a sensitivity of 65.00% and a specificity of 60.00%, with an AUC of 0.575, *p* = 0.321, and a cut-off value of 34.80.

The most accurate prediction of mortality was mNUTRIC, which exhibited an AUC of 0.742, *p* = 0.0003, a cut-off value of 7.50, and sensitivity of 80.20% and specificity of 68.30%.

Among the evaluated nutritional scores, the mNUTRIC score demonstrated the highest discriminative performance for ICU mortality, as reflected by the largest AUC compared with PNI and CONUT.

### 3.8. Survival Analysis

Survival analysis was performed to evaluate the prognostic impact of nutritional risk scores on ICU mortality among patients with sepsis. Time-to-event was defined as the interval between ICU admission and death or censoring at the end of the observation period.

Kaplan–Meier survival curves were constructed to compare survival probabilities between nutritional risk categories (mNUTRIC, PNI, and CONUT), and differences between groups were assessed using the log-rank test. In addition, Cox proportional hazards regression analysis was performed to investigate the association between nutritional scores and mortality risk.

**Figure 4:**
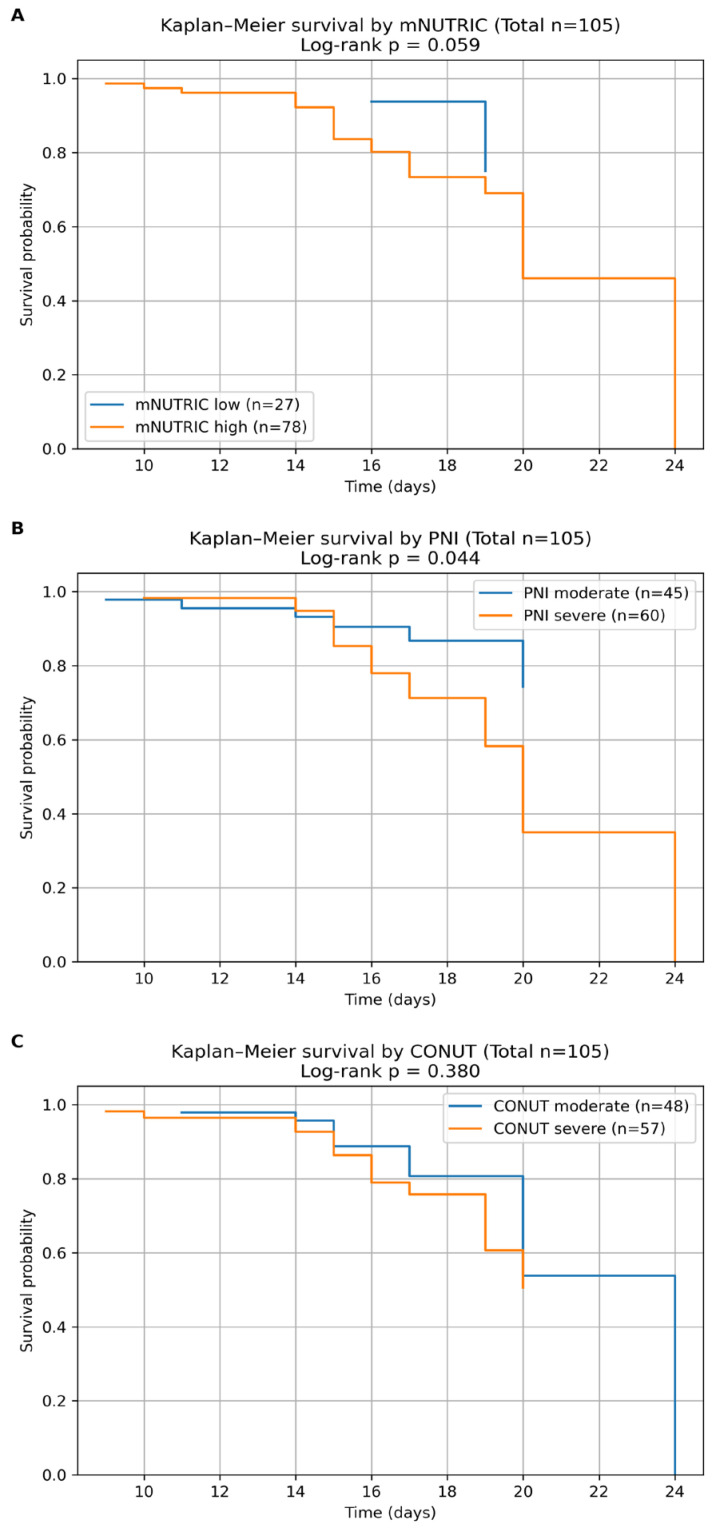
Kaplan–Meier survival curves according to nutritional risk scores (mNUTRIC, PNI, and CONUT) in septic ICU patients. Kaplan–Meier curves illustrate survival probabilities for patients with sepsis admitted to the intensive care unit, stratified by nutritional risk categories defined by the mNUTRIC, PNI, and CONUT scores. Time-to-event was defined as the interval from ICU admission to death or censoring during the observation period. Differences between groups were assessed using the log-rank test. (**A**) Kaplan–Meier survival curves by mNUTRIC category (low vs. high nutritional risk). A trend toward lower survival probability was observed in patients with high mNUTRIC scores (log-rank *p* = 0.059). (**B**) Survival curves by PNI category, comparing moderate and severe malnutrition. Patients with severe malnutrition demonstrated significantly reduced survival probability compared with those with moderate nutritional status (log-rank *p* = 0.044). (**C**) Survival curves by CONUT category, comparing moderate and severe malnutrition. No significant difference in survival probability was observed between CONUT categories (log-rank *p* = 0.380). Numbers in parentheses indicate the number of patients in each category.

Patients with severe malnutrition, as defined by the PNI score, exhibited significantly reduced survival compared with those with moderate nutritional status (log-rank *p* = 0.044) (Table 7 and Figure 4B).

Patients with high nutritional risk, as defined by the mNUTRIC score (Table 7 and Figure 4A), showed a trend toward reduced survival probability compared with those classified as low nutritional risk, approaching statistical significance (log-rank *p* = 0.059). The CONUT score did not significantly discriminate between survival categories in nutritional groups (log-rank *p* = 0.380) (Table 7 and Figure 4C).

These findings suggest that nutritional impairment is associated with reduced survival probability in septic ICU patients, with the strongest discrimination observed for the PNI score in Kaplan–Meier analysis.

In univariate Cox proportional hazards regression analysis (Table 8), the mNUTRIC score was significantly associated with ICU mortality (HR 1.67, 95% CI 1.17–2.38, *p* = 0.005), indicating that higher mNUTRIC values were associated with an increased risk of death in septic patients. Because age, APACHE II, and SOFA scores are structural components of the mNUTRIC index, these variables were not included in multivariate models to avoid structural collinearity.

In an adjusted Cox proportional hazards model including mNUTRIC, sex, and mechanical ventilation (Table 9), the mNUTRIC score showed a trend toward association with ICU mortality (HR 1.39, 95% CI 0.95–2.04, *p* = 0.087). Mechanical ventilation was independently associated with mortality (HR 3.30, 95% CI 1.38–7.88, *p* = 0.007), while sex was not significantly associated with outcome.

## 4. Discussion

The present study evaluated the prognostic association of three nutritional risk scores—mNUTRIC, PNI, and CONUT—with ICU mortality in septic patients with confirmed nutritional risk. In this selected cohort of 105 septic patients and 24 mortality events, mNUTRIC showed the strongest univariate prognostic association with ICU mortality in Cox proportional hazards regression analysis. This finding should be interpreted cautiously: given the small number of events, the pre-selected high-risk cohort, and the composite structure of mNUTRIC (which incorporates APACHE II and SOFA), these results are hypothesis-generating rather than confirmatory. The PNI score significantly discriminated survival probability in Kaplan–Meier analysis, whereas the CONUT score did not discriminate survival outcomes in this cohort.

Beyond the univariate analysis, the multivariable Cox regression model provided additional insight into the prognostic role of the mNUTRIC score. After adjustment for sex and mechanical ventilation—variables not included in the mNUTRIC formula—the association between mNUTRIC and ICU mortality showed a non-significant trend (HR 1.39, 95% CI 0.95–2.04, *p* = 0.087), while mechanical ventilation remained independently associated with mortality (HR 3.30, 95% CI 1.38–7.88, *p* = 0.007). This finding suggests that part of the prognostic signal captured by mNUTRIC may overlap with markers of disease severity and the need for organ support. Importantly, it also highlights the complex interplay between nutritional risk and the severity of critical illness in septic patients.

Considering that sepsis, especially in malnourished patients, is a life-threatening condition, we proposed to study the complex associations between malnutrition and clinical outcomes in critically ill septic patients admitted to the ICU, a condition known for its severe consequences.

The nutritional and disease severity scores provided important insights into the serious risks these patients face regarding their ICU and hospital length of stay and the need for mechanical ventilation.

It should be noted that all these scores are based on investigations that represent first-line tests in the ICU, are easily performed routinely, and have a rapid response time, and they can be valuable tools for early diagnosis, monitoring the patient’s condition, and treatment.

In this study, with a mean age of 62.5 (SD ± 11.7), no statistically significant differences were observed in age between sepsis and non-sepsis patients in the ICU, although age is considered an independent risk factor for mortality and morbidity, especially in critically ill older patients, particularly those with malnutrition, in some studies [21,35]. In contrast, a statistically significant difference was noted in the ages of surviving and non-surviving patients, with 62.40 vs. 68.88 years (*p* < 0.05). Considering the results of our study, we may conclude that age is an important but not independent risk factor for death, with comorbidities playing a significant role.

More than 90% of the critically ill patients with sepsis in our study had more than one comorbidity, particularly diabetes mellitus, hypertension, moderate or severe renal disease, myocardial dysfunction, malignancy (without hematological malignancies), and hemodynamic instability, with many having two or more of these conditions.

The presence of comorbidities associated with malnutrition exacerbates sepsis and may have unfavorable consequences for clinical outcomes, as some previous studies have suggested [21,36]. We considered that the cumulative impact of multimorbidities affects sepsis outcomes, along with disease severity. Thus, we observed a statistically significant difference in the risk of death in patients with multimorbidities (survivor vs. non-survivor, *p* < 0.05).

The two groups, septic and non-septic patients, differ in terms of median length of stay in ICU and hospital: LOS-ICU [8 (4–15) vs. 5 (3–10)] and LOS-H [16 (9–24) vs. 8 (3–20)], which was significantly higher in the septic group vs. the non-septic group, probably also due to the presence of comorbidities, but the death rate was not influenced by LOS-ICU or LOS-H. Our data were comparable to data from other studies, which showed that critically ill patients with sepsis had a longer hospital stay than those without a septic component [37,38].

In our study, we evaluated clinical status, mortality risk, and disease severity using the APACHE II and SOFA scores during the first 24 h of admission and at 72 h. In previous studies of patients with severe sepsis, APACHE II and SOFA scores indicate that the APACHE II score on day 3 is a very good predictor of death [39,40]. According to other authors, Desai S et al., the SOFA score on day three would be a more reliable indicator of mortality [41]. We note that some of these studies were conducted exclusively on the SOFA score or the APACHE II score, and some studies consider that both scores have a favorable predictive value of mortality. We consider that the mandatory use of both scores, in a dynamic manner, is necessary for excellent predictive accuracy in mortality.

Moreover, the prediction of negative outcomes for these scores—APACHE II and SOFA—showed a statistically significant difference (*p* < 0.0001) on both the first and third days. These scores showed good ability to predict mortality (*p* < 0.05), with an AUC of 0.698 for APACHE II and 0.633 for SOFA, along with a sensitivity of 67.90% and specificity of 70.80% for APACHE II and a sensitivity of 63% and specificity of 66.70% for SOFA.

Our study’s primary goal was to assess nutritional status and its relationship to adverse clinical outcomes in critically ill patients, such as mortality, the need for MV, and longer ICU stays. We focused on malnutrition because it is frequently overlooked in sepsis, although it could have a major impact on outcomes in patients with sepsis, particularly those with multimorbidities, by increasing the intensity of systemic inflammation and immunosuppression [42].

The nutritional status on the first day, according to PNI, revealed severe malnutrition risk in 61.90% of the septic population, while according to mNUTRIC and CONUT scores, the risk of severe malnutrition was 74.28% and 54.28%, respectively. We noted statistically significant differences in these scores between the septic and non-septic groups, both on day 1 of admission and on day 3.

Comparing nutritional status between survivor and non-survivor patients, we note, according to PNI, a severe risk of malnutrition with a significant difference between the groups (*p* < 0.05). Similarly, mNUTRIC also indicated a high risk of malnutrition, with a significant difference between the survivor and non-survivor groups (*p* < 0.05). According to the CONUT score, there was no statistically significant difference between the survivor and non-survivor groups (*p* > 0.05).

The connection between clinical factors (such as lab results, age, length of stay in the ICU, APACHE II, and SOFA score) on days 1 and 3 and the three scores studied showed that the mNUTRIC score was much more closely linked to these clinical factors on day 3 of ICU admission. We also noticed a weak negative correlation between the mNUTRIC and PNI values, but not with the CONUT score.

We used ROC curve analysis to assess how well nutritional scores predict outcomes such as survival and ICU hospital stay. We determined a cutoff value for each nutritional score by maximizing the sum of sensitivity and specificity.

According to mNUTRIC, a good prediction of clinical outcomes was noted on both days, day 1 and day 3 of ICU admission, with an AUC of 0.730 (*p* < 0.0001) and an AUC of 0.690 (*p* = 0.0001), respectively, with a cut-off value of 5.50 and 4.50 on the third day, respectively.

Septic patients at high risk of malnutrition, as assessed by their mNUTRIC scores on both day 1 and day 3, had much higher APACHE II and SOFA scores, which were statistically significant (*p* < 0.0001 for both). Also, malnourished (mNUTRIC score) septic patients with mNUTRIC-high risk had substantially higher median LOS-ICU (*p* < 0.05) and LOS-H (*p* < 0.05) and a higher need for MV, with statistically significant differences (*p* > 0.0001). The AUC of the m-NUTRIC score was 0.742 (*p* = 0.0003), and with a cut-off of 7.50, it had an excellent ability to predict 28-day mortality, with a sensitivity of 80.20%. These results are consistent with recent studies showing that the mNUTRIC score is an excellent predictor of 28-day mortality in patients [43,44]. Also, a meta-analysis conducted by Ibrahim DA et al. on 4076 critically ill patients showed that an mNUTRIC score > 5 is associated with an increased risk of death at 28 days, as well as an increase in LOS-ICU [45]. With a cut-off of 7.5 for predicting mortality in our study, we are closer to the results of Jeong DH et al., who reported that the best cut-off of the mNUTRIC score for predicting mortality in sepsis was 6 [46]. Differences may also arise from studying different population groups and therapeutic interventions.

According to the PNI score, there were differences in age and laboratory parameters (*p* < 0.05). Septic patients were associated with significantly higher APACHE II and SOFA values, indicating more severe disease forms (*p* < 0.05 for both).

Additionally, septic patients with severe PNI had substantially higher median LOS-ICU (*p* < 0.05) and LOS-H (*p* < 0.05), and the need for MV was significantly more frequent (*p* = 0.0001). This effect was noticeable only on the first day of evaluation, as on the third day of evaluation of septic patients, statistically significant differences were observed only in age, laboratory parameters, and the need for MV (*p* < 0.05). On day 1 of ICU admission, PNI exhibited a sensitivity of 60% and a specificity of 56%, with an AUC of 0.575, *p* < 0.05, and a cut-off value of 37.30. Our study’s dynamic evaluation revealed that, while the results were consistent with other research on the first day, the predictive capacity of the PNI score could no longer be relied on after day three. The results of previous studies show a strong correlation between PNI scores and clinical outcome prediction, which may be due to the fact that nutritional scores were calculated only on the first day of admission.

The CONUT score in septic patients showed statistically significant differences only in laboratory parameters on the first day (*p* < 0.05). On the third day, age and the requirement for MV were added (*p* < 0.05). We also note that with an AUC of 0.514, a sensitivity of 55.60%, and a specificity of 54.20%, *p* = 0.636, the CONUT score is not a strong predictor of mortality in our study. Thus, our study found that the CONUT score is not consistently usable for predicting outcomes in this category of patients, despite previous studies reporting a significant predictive value, particularly among critically ill patients receiving mechanical ventilation [47]. The limited predictive capacity of the CONUT score may be attributable to serum albumin, which is used in its calculation, given its low sensitivity and specificity for predicting adverse clinical outcomes in critically ill patients [48].

Moghaddam et al.’s study examined five nutritional scores for predicting mortality, organ failure, and MV, with the highest values for NRS-2002 and m-NUTRIC scores [49]. These results confirm our findings regarding m-NUTRIC, PNI, and CONUT scores. This difference between studies is likely because of complicated relationships with other comorbidities, which lead to problems in multiorgan dysfunction and ongoing decline in nutritional status.

Previous studies have reported stronger prognostic performance for mNUTRIC compared with albumin-based indices in ICU populations [42,43], a pattern consistent with the findings of the present cohort. However, a central interpretive consideration must be stated explicitly: mNUTRIC is a composite score that incorporates APACHE II, SOFA, age, number of comorbidities, and pre-ICU hospital stay as structural components. Its stronger prognostic association with ICU mortality in this study may therefore reflect the embedded weight of validated severity-of-illness variables rather than an independent nutritional effect per se. This is not merely a methodological limitation; it is a fundamental issue in interpreting whether mNUTRIC captures nutritional risk, disease severity, or both. The finding that mNUTRIC showed the strongest association with mortality in this selected cohort should accordingly be framed as a cohort-specific observation rather than evidence of definitive clinical superiority over PNI or CONUT as nutritional screening tools. Larger, unselected multicenter studies using time-dependent concordance statistics would be required to disentangle these contributions.

It is important to address the finding that serum albumin was lower in septic compared to non-septic patients. This pattern reflects the well-documented acute-phase response in sepsis: albumin acts as a negative acute-phase reactant, and its plasma concentration decreases rapidly in response to systemic inflammation, hepatic reprioritization of protein synthesis, and capillary leak. This observation should therefore be interpreted primarily as a marker of inflammatory severity rather than chronic nutritional depletion. No patient in this study received exogenous albumin supplementation between Day 1 and Day 3 assessments, confirming that all albumin values represent the endogenous response to sepsis. This distinction is clinically relevant: tools such as PNI and CONUT that incorporate albumin may partly reflect disease severity in the acute sepsis context, which may explain their lower discriminatory capacity compared to mNUTRIC in this cohort.

### 4.1. Prognostic Significance of Nutritional Risk Scores: Survival Analysis and Comparison with Recent Literature

These findings should be interpreted in light of the multivariable Cox regression results, which suggest partial overlap between mNUTRIC and severity-related factors such as mechanical ventilation.

The ROC analysis further supports this finding, as the mNUTRIC score demonstrated the highest discriminative ability among the evaluated nutritional indices. This suggests that mNUTRIC may provide more accurate prognostic stratification than PNI and CONUT in septic ICU patients. Although a formal statistical comparison of AUCs was not performed, the consistent superiority of mNUTRIC across analyses suggests more robust discriminative performance than PNI and CONUT.

The prognostic performance of the mNUTRIC score observed in our study is consistent with its original design as a tool specifically developed to identify critically ill patients at increased nutritional and clinical risk. In our cohort, higher mNUTRIC values were associated with a significantly increased hazard of death, highlighting the relevance of this score as an integrated indicator of disease severity and nutritional vulnerability in septic patients. Importantly, the mNUTRIC score incorporates variables reflecting both physiological severity and comorbidity burden, which likely explains its stronger association with mortality compared with purely nutritional indices. The prognostic value of the mNUTRIC score observed in our study is consistent with recent investigations conducted in critically ill populations. Several contemporary studies have demonstrated that higher NUTRIC or mNUTRIC scores are strongly associated with increased ICU mortality in septic patients. For example, a recent cohort study of septic ICU patients reported that each one-point increase in the NUTRIC score was significantly associated with a higher risk of ICU death, highlighting the score’s role in early risk stratification [50]. Similarly, more recent analyses comparing different nutritional risk indices in septic patients have shown that mNUTRIC often outperforms traditional nutritional scores in predicting mortality, likely because it incorporates both indicators of disease severity and baseline patient vulnerability [51].

In KM survival analysis, patients with severe malnutrition, as defined by the PNI score, exhibited significantly lower survival probabilities than those with moderate nutritional status. The PNI is calculated using serum albumin levels and lymphocyte counts, both of which are markers of systemic inflammation and immune competence. In the context of sepsis, these parameters may reflect the combined impact of nutritional depletion and inflammatory response, which could explain the observed differences in survival between nutritional categories. Our findings regarding the PNI are also supported by recent studies suggesting that this score may reflect both nutritional and immunological status. PNI is derived from serum albumin levels and lymphocyte counts, two parameters closely related to systemic inflammation and immune competence. Recent investigations in septic populations have demonstrated that lower PNI values are associated with increased short-term mortality, particularly in patients with septic shock or those requiring mechanical ventilation [47,52].

The observed differences between KM and Cox regression findings may partly be explained by the methodological approaches used in each analysis. The KM curves were constructed using categorized variables, which may lead to loss of information and reduced statistical power. In contrast, Cox proportional hazards regression preserves the continuous nature of the predictors and may therefore provide a more sensitive and accurate estimation of their association with mortality risk. This may explain why the PNI score showed statistical significance in survival curve analysis, while the mNUTRIC score demonstrated a stronger prognostic signal in Cox regression and ROC analyses.

In contrast, the CONUT score did not significantly discriminate between survival rates across nutritional categories in our population. Although the CONUT index includes albumin, total cholesterol, and lymphocyte count as indicators of nutritional status, its ability to predict outcomes may be affected by the metabolic changes typical of critical illness. In septic patients, rapid changes in lipid metabolism and acute inflammatory responses may limit the ability of cholesterol-based indices to accurately reflect nutritional risk. In contrast, the CONUT score did not significantly discriminate survival in our cohort. Although CONUT incorporates albumin, cholesterol, and lymphocyte count, several recent studies suggest that cholesterol-based nutritional indices may have limited prognostic accuracy in critically ill patients. Acute inflammatory responses and metabolic alterations during sepsis can substantially modify lipid metabolism, potentially reducing the reliability of cholesterol-derived nutritional markers in predicting clinical outcomes [47].

Taken together, these findings suggest that while traditional nutritional indices such as PNI and CONUT provide useful information regarding nutritional and immunological status, scores specifically developed for critically ill populations, such as mNUTRIC, may provide a more robust prognostic assessment in septic ICU patients.

### 4.2. Study Limitations

Several limitations of this study should be acknowledged. First, although data were collected from two ICUs (Clinical Municipal Hospital “Filantropia” Craiova and the Emergency County Clinical Hospital of Craiova), the study remains regionally limited, and findings may not be generalizable to other ICU populations or healthcare settings with different case-mix or antimicrobial resistance profiles. The sample size was relatively modest, with only 24 mortality events in the septic cohort, substantially limiting statistical power; findings should be considered exploratory. The inclusion criterion requiring all three scores to be simultaneously met introduces a selection effect: enrolled patients represent a high-risk nutritional subgroup, and score distributions may differ materially from those in unselected ICU populations. Exclusion of patients without available proxy consent may have introduced selection toward less severely ill cases. Day-3 reassessments are vulnerable to survivor bias, since only patients remaining in the ICU at 72 h were reassessed.

Second, the sample size was relatively modest, particularly with respect to the number of mortality events, which may have limited statistical power to detect associations between some nutritional indices and survival outcomes.

Third, nutritional scores were calculated at the time of ICU admission, and dynamic changes in nutritional status during the ICU stay were not evaluated. Because nutritional and inflammatory parameters may evolve rapidly during sepsis, longitudinal assessment of these indices could provide additional prognostic information. Additionally, NRS-2002 (Nutritional Risk Screening 2002), BMI, and weight-loss history were not routinely recorded during the study period and could not be incorporated into the nutritional assessment. These parameters, recommended by ESPEN guidelines, would provide complementary nutritional context, and their absence represents a limitation that future studies should address. The use of serum albumin as a nutritional marker in the acute sepsis setting is further limited by its role as a negative acute-phase reactant, which may confound the interpretation of PNI and CONUT.

Finally, although several clinically relevant variables were included in the analysis, residual confounding cannot be completely excluded, as this is inherent to observational studies.

Importantly, these findings apply specifically to a selected cohort of septic ICU patients with confirmed nutritional risk and should not be generalized to the broader population of critically ill septic patients.

## 5. Conclusions

In this prospective observational cohort of septic ICU patients with confirmed nutritional risk, nutritional scoring was evaluated as a prognostic approach for mortality. Among the investigated indices, mNUTRIC demonstrated the strongest univariate prognostic signal for ICU mortality; however, this association was attenuated and did not reach statistical significance after limited multivariable adjustment. Kaplan–Meier analysis further showed that severe malnutrition, as defined by the PNI score, was associated with significantly reduced survival probability, whereas the CONUT score did not discriminate survival outcomes in this cohort. These findings should be interpreted with caution, as they derive from a selected cohort with a limited number of events. The stronger prognostic signal observed for mNUTRIC likely reflects not only nutritional risk but also its embedded components of disease severity, including APACHE II and SOFA scores. Overall, our results support the clinical relevance of early nutritional risk assessment in septic ICU patients while emphasizing that different tools may capture overlapping but distinct aspects of patient risk; however, these findings should be interpreted as exploratory and specific to a selected cohort of patients with confirmed nutritional risk and should not be generalized to all critically ill septic patients. Further large-scale, multicenter studies incorporating time-dependent analyses and appropriately adjusted multivariable models are needed to better define the independent contribution of nutritional status to outcomes in sepsis.

## Figures and Tables

**Table 1:** Demographic parameters of patients grouped by the study categories on day 1.

Parameters		S Group Day 1 (*n* = 105)	NS Group Day 1 (*n* = 50)	*p*-Value from Pearson’s Chi-Squared/ Student’s *t*-Test
Age (years)	Mean ± SD	62.56 ± 11.78	59.74 ± 14.30	0.318
Gender, *n*	Male/Female	65/40	23/27	0.062
Residence, *n*	Urban/Rural	54/51	36/14	0.015
*Severity of the patient’s disease*				
APACHE II score	Median (range)	28 (15–44)	18 (8–35)	<0.0001
SOFA score	Median (range)	9 (4–13)	7 (2–11)	<0.0001
*Comorbidity, n*				
Endocrine (Diabetes mellitus)		26	19	0.018
Vascular (Hypertension)		41	20	0.377
Myocardial (Angina, Arrhythmia, Heart disease, MI)		41	29	0.001
Pulmonary (Asthma, COPD)		21	15	0.055 ^#^
Malignancy (without hematological malignancy)		23	12	0.416
Renal (moderate or severe renal disease)		13	13	0.019
Neurologic (Dementia, Neurologic illnesses)		19	10	0.458
Musculoskeletal (Arthritis, Osteoporosis, Connective tissue disease)		36	20	0.152
Hemodynamic instability		34	14	0.877
*Reasons for ICU admission/Sepsis-Source of infection*				
Respiratory System Disorders		26	8	0.312
Trauma		15	5	0.681
Gastrointestinal System Disorders		33	17	0.751
Urogenital System Disorders		31	20	0.195
*Number of comorbidities, n (%)*				
0–1		8	14	0.0006
2 or multiple		98	36	
*Laboratory parameters*				
Hb (g/dL)	Mean	11.16	10.90	0.577
	±SD	2.60	2.78	
Ht (%)	Mean	32.49	33.77	0.387
	±SD	9.35	8.14	
WBC (×10^3^/μL)	Median	9.30	8.99	0.053 ^#^
	range	3.42–38.40	5.62–21.40	
LYM (×10^3^/μL)	Median	1.47	0.90	0.0006
	range	0.10–4.97	0.17–6.03	
PLT (×10^3^/μL)	Median	245	178	<0.0001
	range	78–308	68–549.5	
PCT (ng/mL)	Median	1.25	0.36	<0.0001
	range	0.23–11.02	0.13–7.23	
ALB (g/dL)	Mean	3.10	3.28	0.038
	±SD	0.57	0.79	
CRP (mg/mL)	Median	91.20	39.70	0.027
	range	16.29–407.0	8.59–474.0	
TC (mmol/L)	Median	128.70	108.50	0.038
	range	87.42–287.60	88.55–210.60	
*Nutritional Status Assessments*				
PNI	Median (range)	38.95 (14.00–55.85)	36.50 (21.50–52.50)	0.345
	Moderate (40–45), *n*	40	19	0.862
	Severe (<40), *n*	65	31	
CONUT	Median (range)	5 (2–12)	6 (2–12)	0.136
	Moderate (5–8), *n*	48	39	0.0002
	Severe (9–12), *n*	57	11	
mNUTRIC	Median (range)	7 (3–9)	5 (1–8)	<0.0001
	Low risk, *n*	27	27	0.011
	High risk, *n*	78	33	
*LOS-ICU*	Median (range)	8 (4–15)	5 (3–10)	<0.0001
*LOS-H*	Median (range)	16 (9–24)	8 (3–20)	<0.0001
*Need for MV, n (%)*		43 (40.95%)	10 (23.25%)	0.041

APACHE II: Acute Physiology and Chronic Health Evaluation II; SOFA: Sequential Organ Failure Assessment; Hb: hemoglobin; Ht: hematocrit; WBC: white blood cells/leukocytes; CRP: C-reactive protein; PLT: platelets; TC: total cholesterol; PCT: procalcitonin; ALB: albumin; LYM: lymphocytes; PNI: Prognostic Nutritional Index; CONUT: Controlling Nutritional Status; mNUTRIC: Nutrition Risk in the Critically Ill; LOS-ICU: length of stay in Intensive Care Unit; LOS-H: length of stay in hospital; MV: mechanical ventilation; MI: myocardial infarction; COPD: chronic obstructive pulmonary disease; ^#^: reaching the significance limit.

**Table 2:** Demographic parameters of the patients stratified according to the groups under study on day 3.

Parameters		S Group Day 3 (*n* = 105)	NS Group Day 3 (*n* = 50)	*p*-Value from Pearson’s/Chi-Squared/ Student’s *t*-Test
*Severity of the patient’s disease*				
APACHE II score	Median (range)	25 (9–43)	16 (8–35)	<0.0001
SOFA score	Median (range)	8 (3–15)	6 (3–17)	0.001
*Laboratory parameters*				
Hb (g/dL)	Mean ± SD	10.86 ± 2.20	10.46 ± 2.06	0.366
Ht (%)	Mean ± SD	32.03 ± 6.28	31.24 ± 7.12	0.400
WBC (×10^3^/μL)	Median	11.80	10.25	0.006
	range	2.70–72.50	3.20–19.90	
LYM (×10^3^/μL)	Median	1.26	0.94	0.046
	range	0.10–7.20	(0.20–2.00)	
PLT (×10^3^/μL)	Median	157.5	236	<0.0001
	range	38–309	34–439	
PCT (ng/mL)	Median	1.10	0.41	0.016
	range	0.14–9.23	0.12–8.83	
ALB (g/dL)	Mean ± SD	2.84 ± 0.43	2.90 ± 0.44	0.427
CRP (mg/mL)	Median	102.2	61.8	0.027
	range	10.3–443.5	3.4–313.2	
TC (mmol/L)	Median	127.60	117.30	0.019
	range	88.12–224.40	87.24–162.20	
*Nutritional Status Assessments*				
PNI	Median (range)	35.30 (22.00–62.00)	34.90 (21.50–41.95)	0.321
	Moderate (40–45), *n*	27	16	0.413
	Severe (<40), *n*	78	34	
CONUT	Median (range)	7 (4–12)	7 (1–12)	0.073
	Moderate (5–8), *n*	44	35	0.001
	Severe (9–12), *n*	61	15	
mNUTRIC	Median (range)	6 (3–9)	5 (1–8)	<0.0001
	Low risk, *n*	47	32	0.025
	High risk, *n*	58	18	
*Need for MV, n (%)*		58 (55.23%)	8 (18.60%)	<0.0001

APACHE II: Acute Physiology and Chronic Health Evaluation II; SOFA: Sequential Organ Failure Assessment; Hb: hemoglobin; Ht: hematocrit; WBC: white blood cells/leukocytes; CRP: C-reactive protein; PLT: platelets; PCT: procalcitonin; TC: total cholesterol; ALB: albumin; LYM: lymphocytes; PNI: Prognostic Nutritional Index; CONUT: Controlling Nutritional Status; mNUTRIC: Nutrition Risk in the Critically Ill; MV: mechanical ventilation; I

**Table 3:** Comparison of clinical parameters between survivor and non-survivor patients.

Parameters		Survivor (*n* = 81)	Non-Survivor (*n* = 24)	*p*-Value from Pearson’s/Chi-Squared/ Fisher/Student’s *t* Test
Age (years)	Mean ± SD	62.40 ± 11.41	68.88 ± 13.86	0.022
Gender, *n*	Male/Female	48/33	17/7	0.305
Residence, *n*	Urban/Rural	45/36	9/15	0.120
*Severity of the patient’s disease*				
APACHE II score	Median (range)	27.00 (15–44)	29.50 (18–37)	0.003
SOFA score	Median (range)	9 (4–13)	10 (4–14)	0.046
*Comorbidity, n*				
Endocrine (Diabetes mellitus)		20	15	<0.0001
Vascular (Hypertension)		31	18	0.001
Myocardial (Angina, Arrhythmia, Heart disease, MI)		26	16	0.002
Pulmonary (Asthma, COPD)		14	7	0.246
Malignancy (without hematological malignancy)		16	7	0.327
Renal (moderate or severe renal disease)		5	8	0.151
Neurologic (Dementia, Neurologic illnesses)		13	6	0.367
Musculoskeletal (Arthritis, Osteoporosis, Connective tissue disease)		23	5	0.462
Hemodynamic instability		21	17	<0.0001
*Reasons for ICU admission/Sepsis-Source of infection, n*				
Respiratory System Disorders		16	12	0.003
Trauma		21	18	<0.0001
Gastrointestinal System Disorders		25	8	0.823
Urogenital System Disorders		24	7	0.590
Bone/soft tissue		2	-	
*Number of comorbidities, n (%)*				
0–1		16	-	
2 or multiple		65	24	0.020
*Laboratory parameters*				
WBC (×10^3^/μL)	Median (range)	8.37 (3.42–20.00)	16.55 (6.96–101.80)	<0.0001
LYM (×10^3^/μL)	Median (range)	1.46 (0.10–3.90)	2.62 (0.30–4.97)	0.019
ALB (g/dL)	Mean ± SD	3.14 ± 0.59	2.06 ± 0.50	0.025
CRP (mg/mL)	Median (range)	85.05 (55.08–380.60)	97.73 (60.10–407.00)	0.038
TC (mmol/L)	Median (range)	126.80 (87.45–220.10)	128.80 (87.42–287.70)	0.666
*Nutritional Status Assessments*				
PNI	Median (range)	39.75 (27.25–55.85)	30.10 (14.00–45.50)	0.014
	Moderate (40–45), *n*	39	6	0.044
	Severe (<40), *n*	42	18	
CONUT	Median (range)	5 (2–12)	6 (2–12)	0.510
	Moderate (5–8), *n*	38	10	0.647
	Severe (9–12), *n*	43	14	
mNUTRIC	Median (range)	6 (3–9)	8 (4–9)	0.0002
	Low risk, *n*	25	2	0.027
	High risk, *n*	56	22	
LOS-ICU	Median (range)	8 (6–15)	6 (4–12)	0.196
LOS-H	Median (range)	15 (11–22)	14 (9–24)	0.361
Need for MV, *n*		15	24	<0.0001

APACHE II: Acute Physiology and Chronic Health Evaluation II; SOFA: Sequential Organ Failure Assessment; WBC: white blood cells/leukocytes; CRP: C-reactive protein; TC: total cholesterol; ALB: albumin; LYM: lymphocytes; PNI: Prognostic Nutritional Index; CONUT: Controlling Nutritional Status; mNUTRIC: Nutrition Risk in the Critically Ill; MV: mechanical ventilation; LOS-ICU: length of stay in Intensive Care Unit; LOS-H: length of stay in hospital; MI: myocardial infarction; COPD: chronic obstructive pulmonary disease.

**Table 4:** Comparison of the PNI, mNUTRIC, and CONUT groups’ clinical parameters on day 1 of ICU admission from the S group.

Characteristics	S Group Day 1 (*n* = 105)								
	PNI			mNUTRIC			CONUT		
	Moderate	Severe	*p*-Value	Low	High	*p*-Value	Moderate	Severe	*p*-Value
Patients (*n*)	40	65		27	78		48	57	
*Demographic characteristics*									
Age (yrs) (mean ± SD)	62.53	65.76	0.024	56.07	66.58	<0.0001	65.23	67.07	0.116
	13.57	11.76		11.43	11.40		12.00	11.48	
Gender, Female/Male (*n*)	16/24	24/41	0.751	8/19	32/46	0.294	20/28	20/37	0.488
Area of residence, Rural/Urban (*n*)	19/21	32/33	0.862	12/15	39/39	0.617	25/23	26/31	0.507
*Medical condition*									
APACHE II score [median (range)]	28	34	0.019	26	30	<0.0001	29	28	0.086
	15–32	17–44		15–27	19–44		18–39	17–44	
SOFA score [median (range)]	9	11	0.044	8	10	<0.0001	10	9	0.148
	4–12	4–13		4–10	4–13		4–13	4–13	
LOS-ICU [median (range)]	8	10	0.046	6	10	0.034	8	9	0.064
	4–13	8–15		4–9	7–15		5–13	6–15	
LOS-H [median (range)]	15	18	0.039	15	17	0.026	16	17	0.068
	9–22	10–24		7–22	10–24		10–20	10–24	
Need for MV, *n* (%)	7	36	0.0001	6	37	0.022	15	28	0.063
*Laboratory parameters*									
WBC (×10^3^/μL) (mean ± SD)	10.20	12.35	0.043	9.75	12.14	0.302	9.68	13.10	0.245
	4.60	10.45		4.57	11.66		3.75	10.72	
LYM (×10^3^/μL) (mean ± SD)	1.68	2.09	0.007	1.26	1.64	0.098	1.53	1.83	<0.0001
	0.77	0.92		0.72	0.89		0.81	0.83	
TC (mmol/L) [median (range)]	132.40	176.40	<0.0001	122.10	129.00	0.158	124.60	177.40	<0.0001
	98.60–220.10	105.80–287.60		87.45–204.60	87.42–287.60		92.30–184.30	102.80–287.60	
ALB (g/dL) (mean ± SD)	3.48	2.72	<0.0001	3.30	3.03	0.131	3.27	2.46	<0.0001
	0.45	0.47		0.75	0.48		0.67	0.47	
CRP (mg/dL) (mean ± SD)	102.10	128.00	0.017	109.50	133.60	0.022	120.80	154.50	0.032
	91.48	83.26		79.38	109.10		64.82	116.70	

APACHE II: Acute Physiology and Chronic Health Evaluation II; SOFA: Sequential Organ Failure Assessment; WBC: white blood cells/leukocytes; CRP: C-reactive protein; TC: total cholesterol; ALB: albumin; LYM: lymphocytes; PNI: Prognostic Nutritional Index; CONUT: Controlling Nutritional Status; mNUTRIC: Nutrition Risk in the Critically Ill; MV: mechanical ventilation; LOS-ICU: length of stay in Intensive Care Unit; LOS-H: length of stay in hospital.

**Table 5:** Comparison of the PNI, mNUTRIC, and CONUT groups’ clinical parameters on day 3 of ICU admission from the S group.

Characteristics	S Group Day 3 (*n* = 106)								
	PNI			mNUTRIC			CONUT		
	Moderate	Severe	*p*-Value	Low	High	*p*-Value	Moderate	Severe	*p*-Value
Patients (*n*)	27	78		47	58		44	61	
*Demographic characteristics*									
Age (yrs) (mean ± SD)	61.67	65.50	0.156	58.28	69.57	<0.0001	63.02	69.87	0.012
	13.36	11.54		11.02	10.50		12.32	9.76	
Gender, Female/Male (*n*)	14/13	27/51	0.113	17/30	24/34	0.583	18/26	23/38	0.740
Area of residence, Rural/Urban (*n*)	10/17	41/37	0.163	24/23	27/31	0.646	21/23	30/31	0.887
*Medical condition*									
APACHE II score [median (range)]	25	25	0.281	25	33	<0.0001	25	27	0.267
	13–32	9–43		9–27	20–43		13–32	9–38	
SOFA score [median (range)]	8	8	0.340	6	10	<0.0001	8	9	0.187
	5–13	3–15		3–9	3–15		3–14	4–15	
LOS-ICU [median (range)]	7	9	0.076	6	10	0.036	8	8	0.501
	6–11	4–15		4–15	6–15		4–15	6–12	
LOS-H [median (range)]	15	17	0.061	14	18	0.016	16	16	0.889
	10–18	12–24		9–22	11–24		9–22	11–24	
Need for MV, *n* (%)	10	48	0.027	14	44	<0.0001	19	39	0.035
*Laboratory parameters*									
WBC (×10^3^/μL) (mean ± SD)	13.39	14.92	0.421	11.37	15.74	0.008	13.76	15.79	0.244
	6.70	11.79		4.17	10.35		11.34	8.20	
LYM (×10^3^/μL) (mean ± SD)	1.06	2.16	<0.0001	1.32	1.37	0.819	1.43	1.78	<0.0001
	0.52	1.32		1.03	0.82		0.76	1.23	
TC (mmol/L) [median (range)]	115.40	148.30	<0.0001	118.60	122.10	0.764	119.30	146.30	<0.0001
	103.20–224.40	88.12–201.60		93.58–210.60	88.12–224.40		92.12–201.60	103.20–224.40	
ALB (g/dL) (mean ± SD)	3.21	2.71	<0.0001	2.95	2.76	0.023	3.14	2.47	<0.0001
	0.37	0.36		0.46	0.38		0.35	0.31	
CRP (mg/dL) (mean ± SD)	110.80	123.50	0.870	120.90	122.20	0.933	126.10	135.80	0.281
	70.04	88.59		59.61	90.82		87.24	86.65	

APACHE II: Acute Physiology and Chronic Health Evaluation II; SOFA: Sequential Organ Failure Assessment; WBC: white blood cells/leukocytes; CRP: C-reactive protein; TC: total cholesterol; ALB: albumin; LYM: lymphocytes; PNI: Prognostic Nutritional Index; CONUT: Controlling Nutritional Status; mNUTRIC: Nutrition Risk in the Critically Ill; MV: mechanical ventilation; LOS-ICU: length of stay in Intensive Care Unit; LOS-H: length of stay in hospital.

**Table 6:** The accuracy of the PNI, mNUTRIC, and CONUT to predict the outcomes of ICU patients.

Parameter	AUC	Std. Error	Cut-Off Values	95% CI	Sensitivity %	Specificity %	Youden Index	*p*-Value
day 1								
APACHE II on day 1	0.804	0.041	24.50	0.723–0.885	82.90	64.00	0.469	<0.0001
SOFA on day 1	0.746	0.045	7.50	0.658–0.834	63.80	60.00	0.238	<0.0001
mNUTRIC on day 1	0.730	0.044	5.50	0.644–0.817	74.30	54.00	0.283	<0.0001
PNI on day 1	0.575	0.050	37.30	0.480–0.670	60.00	56.00	0.160	0.035
CONUT on day 1	0.546	0.048	6.50	0.449–0.644	53.30	56.00	0.093	0.131
day 3								
APACHE II on day 3	0.785	0.046	22.50	0.695–0.875	96.20	60.00	0562	<0.0001
SOFA on day 3	0.691	0.054	6.50	0.583–0.796	86.70	62.00	0.487	0.0001
mNUTRIC on day 3	0.690	0.051	4.50	0.591–0.789	70.00	66.00	0.360	0.0001
PNI on day 3	0.575	0.058	34.80	0.443–0.698	51.40	50.00	0.014	0.321
CONUT on day 3	0.549	0.048	7.50	0.455–0.644	61.90	56.00	0.179	0.130
non-survivor								
mNUTRIC	0.742	0.059	7.50	0.627–0.857	80.20	68.30	0.485	0.0003
APACHE II	0.698	0.067	28.50	0.567–0.829	67.90	70.80	0.387	0.003
SOFA	0.633	0.076	9.50	0.484–0.781	63.00	66.70	0.297	0.049
PNI	0.532	0.071	38.80	0.392–0.672	53.10	50.00	0.031	0.831
CONUT	0.514	0.072	5.50	0.373–0.653	55.60	54.20	0.098	0.636

**Table 7:** Kaplan–Meier survival analysis by nutritional risk categories (mNUTRIC, PNI, and CONUT).

Nutritional Score	Category	Number of Patients (*n*)	Deaths (*n*)	Log-Rank *p*-Value
mNUTRIC	Low risk	27	2	0.059
	High risk	78	22	
PNI	Moderate malnutrition	45	7	0.044
	Severe malnutrition	60	17	
CONUT	Moderate malnutrition	48	10	0.380
	Severe malnutrition	57	14	

**Table 8:** Univariate Cox proportional hazards regression analysis for predictors of ICU mortality in septic patients.

Variable	Hazard Ratio (HR)	95% CI	*p*-Value
mNUTRIC	1.67	1.17–2.38	0.005
PNI	0.99	0.93–1.05	0.767
CONUT	1.05	0.91–1.22	0.504
Age	1.04	1.00–1.08	0.067
APACHE II	1.09	0.99–1.20	0.096
SOFA	1.25	0.99–1.58	0.058

CI: confidence interval. Hazard ratios were estimated using univariate Cox proportional hazards regression models.

**Table 9:** Multivariable Cox proportional hazards regression analysis including mNUTRIC, sex, and mechanical ventilation in septic ICU patients.

Variable	Hazard Ratio (HR)	95% CI	*p*-Value
mNUTRIC	1.39	0.95–2.04	0.087
Sex	1.32	0.53–3.28	0.556
Mechanical ventilation	3.30	1.38–7.88	0.007

CI: confidence interval.

## Data Availability

The data presented in this study are available on request from the corresponding author due to ethical reasons.
